# Women’s Perceived Barriers to Accessing Post-Abortion Care Services in Selected Districts in KwaZulu Natal Province, South Africa: A Qualitative Study

**DOI:** 10.5334/aogh.3888

**Published:** 2022-08-26

**Authors:** M. Netshinombelo, M. S. Maputle, D. U. Ramathuba

**Affiliations:** 1 Department of Advanced Nursing, University of Venda, South Africa. Private Bag X5050, Thohoyandou, 0950, South Africa

**Keywords:** Accessibility, barriers experienced, post-abortion care, post-abortion complications

## Abstract

**Background:**

Despite different interventions to curb unwanted pregnancies, repositioning Family Planning and post-abortion care services as critical priorities in health programme in Kwa-Zulu Natal, women are still admitted with induced abortion complications.

**Aim:**

The aim was to explore challenges experienced by women who are accessing post-abortion care services at selected public hospitals.

**Methods:**

A qualitative explorative, descriptive, and contextual approach was used. The population comprised all women who presented with incomplete abortion and had accessed PAC services in the selected hospitals. Purposive convenience sampling was used to select the 23 participants. Data were collected through in-depth interviews with all participants on discharge and analysed through open-coding method. Trustworthiness was ensured, by considering the criteria of credibility, dependability, applicability, and transferability. Ethical considerations were secured by requesting consent and permission to conduct the study. All interviews were voluntarily conducted following the ethically approved informed consent, anonymity and confidentiality were maintained.

**Findings:**

Three themes emerged reflecting challenges from women’s perspectives on accessing post abortion care. These themes were: transportation barriers, stigma, and mistreatment (lack of analgesics, early discharge).

**Conclusion:**

Conclusion showed the description of perceived barriers and experiences related to accessing, seeking and care received during the process of PAC. Inaccessibility to PAC was due to poor road, poor mode, lack of transport and long distance from the community to the hospital, long waiting queues, stigma, and discrimination. The PAC services should be accessible with the increased number of facilities and adequately trained staff with functional equipment and guidelines. Value clarification workshops for health professionals are essential.

## Introduction

The WHO defines abortion as the termination of a pregnancy, whether spontaneous or induced, before 22 weeks of gestation [1]. Unsafe abortion is defined as any procedure to terminate a pregnancy by persons lacking the proper skills and/or is performed in an unclean, non-medical setting [1]. Complications of spontaneous or induced abortion are the fourth leading direct cause of maternal mortality globally [2]. Post Abortion Care (PAC) needs to be introduced effectively to address these complications. PAC is a package of services offered to women after an incomplete abortion due to spontaneous or induced abortion [3]. PAC focuses on five primary elements, namely: treatment and management of incomplete abortion and complications, counseling, contraceptive and family planning services, reproductive and other health services, and community and service provider partnerships to prevent complications of unwanted pregnancies and unsafe abortion [4]. The procedures and technologies used includes manual/electrical vacuum aspiration (M/EVA), medication abortion (MA) drugs (Misoprostol and Mifepristone), digital (use of bare fingers) evacuation, forceps, and dilatation and curettage [5]. PAC is a package of interventions that has been shown to reduce abortion-related morbidity and mortality when available and of good quality [6]. Women in South Africa are affected mainly by unwanted pregnancies, which lead to illegal abortion [7]. A research study by Koster revealed that 12% of abortions reported were undoubtedly illegally induced [8]. The study further revealed three factors possibly associated with women having induced abortions. The majority of women report that their pregnancy was unwanted, 30.4% reported that they were not married or had extramarital sex and became pregnant.

Women experience barriers when needing to access the PAC services [9]. Lack of access to abortion services leaves women and young girls no other choice than unsafe abortion, thus placing a tremendous burden on the South African health system and making unsafe abortion one of the major contributors to maternal mortality in South Africa including KwaZulu Natal [10]. Most rural areas and many primary health centres do not offer any emergency stabilization or intervention for women with abortion complications before referral to the next level of care. Decentralization of PAC services are essential, bringing immediate life-saving care and preventing unnecessary deterioration of the woman’s condition when referral and transport are required. Women from rural areas travel long distances to access PAC services compared to women from urban areas. The long-distance estimated from the rural to urban area is 300 km in case of emergency when a woman needs tertiary service. One of the main challenges for women who access PAC services in KZN is the long distances they have to travel to access proper care in the hospital; poor roads and transportation further complicate this in their areas. This was corroborated by Perera et al. that delays in care-seeking due to various reasons, including lack of transport and concerns about costs [6]. Literature shows that increased distance to health facilities and poor local transportation impedes accessibility to the primary and secondary health care services as well as that the delayed health care provision can worsen people’s health [11,12]. Post-abortion care services should be available and provided to all women who have suffered from complications of abortion. Women and health care workers play a significant role in providing post-abortion care services, however, their religious and cultural beliefs play an essential role in the provision of safe abortion and post-abortion care [13].

South Africa legalized abortion in 1996 through the Choice in Termination of Pregnancy Act [14]. Despite the legalization of abortion, the negative attitudes and judgemental behaviours surrounding abortion are enormous and call for urgent actions. Women reported stigma, from health care workers as what keeps them away from the facility and drives them to seek abortion services from traditional birth attendants and bogus doctors who offer unsafe abortions that lead to abortion-related complications and increasing morbidity and mortality [9]. A study by Gebreselassie found that women tend to be reluctant to seek post abortion care due to fear of ill treatment from the health care workers, especially if they have undergone an illegal abortion [15]. Shellenberg et al. and Adee et al. reported that two out of three women experienced stigma following abortion, hence most women prefer to keep their abortion secret [9,16].

Women’s access to abortion is determined by legal restrictions that vary across the country and the geographic availability of abortion clinics. In South Africa, new restrictions against the procedure were introduced in Choice on Termination of Pregnancy (CTOP Act), 1996 [14]. According to CTOPA, a woman can procure an abortion under the following conditions: (1) Within the first 12 weeks of gestation, a woman may terminate her pregnancy on demand. (2) Between 13 and 20 weeks gestation, a woman may terminate her pregnancy if her medical practitioner, after having consulted with the patient, determines that her pregnancy will threaten her mental or physical health; the fetus may be subjected to acute physical or mental disability; pregnancy arose out of rape or incest; or her pregnancy will detrimentally affect her socioeconomic standing. (3) After 20 weeks gestation, a woman may only terminate her pregnancy if her medical practitioner, after consulting with another medical practitioner or registered midwife, determines that the patient’s life is in jeopardy; there is acute malformation of the fetus; or the fetus could be significantly injured during delivery. Most of these restrictions severely curtail women’s access to the procedure by limiting women’s ability to obtain the procedure and physicians’ or clinics’ ability to offer it [17]. Additional restrictions that do not allow medical practitioners, without accreditation, are in place to discourage providers from opening the clinics and that makes it more difficult for women to access the procedure. Following the accessing of the procedure may accompanied by psychological trauma. Despite different interventions to curb unwanted pregnancies and repositioning of Family Planning (FP) as a critical priority in health programme in KwaZulu Natal (KZN), women are still admitted with induced abortion complications, presumably resulting from unsafe procedures outside these district hospitals [18]. KwaZulu Natal Districts Health Information System: 2018/19 statistics (April 2018 to March 2019) indicated that 4 869 women experienced incomplete abortions and 183 septic abortions, while the rural areas experienced 2 541 incomplete abortions and 103 septic abortions respectively. Furthermore, women who experience complications also faced challenges in accessing post-abortion care.

The results of unsafe abortion complications depended on the accessibility and quality of PAC services and implementation of guidelines and the capability of women to seek care [19]. This study aimed to explore challenges experienced by women who were accessing PAC services at selected districts of KZN.

## Methods

A qualitative approach and explorative, descriptive, and contextual designs were used. The study was conducted in 2019 at 23 hospitals in five selected districts in KwaZulu-Natal: eThekwini, uMgungundlovu, Harry Gwala, uMzinyathi and King Cetswayo (as a subsection of the Ph.D. study) [20]. The five districts were selected because of incomplete and septic abortions statistics.

### Population and sampling

The population comprised all women who presented with incomplete abortion and had accessed PAC services in the selected public hospitals. Twenty-three women, one from each hospital, agreed to participate and were purposely sampled. Women were recruited for interviews by the researcher on discharge. Convenience sampling was employed, and women were recruited irrespective of age and those who refused to participate were excluded from the study. All hospitals in the province that were providing PAC were included in the study and a total of 23 hospitals were purposively sampled.

### Ethical considerations

Ethical standards were ensured by obtaining the ethical clearance (Ref: SHS/17/PDC/15/1808), from the University of Ethics Committee, permission to conduct the study from the KZN Provincial Department of Health, the hospital Chief Executive Officer, unit managers, and the participants. Ethical principles of informed consent, privacy, anonymity, confidentiality, and rights to self-determination were adhered to. Participants signed informed consent before participating. The data collection was done in a separate room to guarantee anonymity, privacy, and confidentiality maintained by reporting the coded findings.

### Data collection

Data were collected through unstructured face-to-face interviews to gain a detailed narrative of participants on the perceived challenges experienced when accessing post-abortion care services. The researcher requested that the unit manager allocate a quiet room to ensure comfort and privacy during the interview. Central opening questions initiated the interview: “*Kindly share with me the barriers you experienced and the support you received when accessing post-abortion care service*.” The interviews were conducted by researchers in the participants’ local language (isiZulu), and each lasted between 30–45 minutes. Permission to use the voice recorder was obtained from participants.

### Data analysis

The narrative data from the in-depth individual interviews were analyzed qualitatively using Tesch’s open coding method as postulated by Creswell [21]. The recorded interviews were translated into English by a language expert. They transcribed word by word, and the nonverbal cues (for example, silence/sigh, frowns, and lean back) were included in the transcripts. All transcripts were read to give meaning, and a list of similar topics clustered. Data were grouped according to categories and sub-categories and field notes were also coded and categorized. A literature control was done to control the results of the study.

### Trustworthiness

As outlined in Polit and Beck [22], the criteria for ensuring trustworthiness were adhered to. Credibility was ensured through prolonged engagement during data collection. The researcher met with the participant to establish rapport and to make an appointment. During the interviews, the researcher spent time with the participants listening to and observing them as they were interviewed. The participants were interviewed to the point at which there was data saturation. A member check was also conducted to validate the truth and confirm the findings. The voice recorder was used to ensure credibility. Transferability was ensured by thick descriptions of the research methodology. The recorded interviews were transcribed verbatim, and the nonverbal cues (for example, silence/sigh, frowns, and lean back) were included in brackets of the transcripts to ensure authenticity. Researchers undertook a qualitative approach to mitigate potential bias and the researcher adhered to the principle of bracketing which is a method used to mitigate the potential deleterious effects of unacknowledged preconceptions related to the research and thereby to increase the rigor of the project.

## Presentation of findings

Table 1 present the demographic profile of participants. In this study, there was 52% of single women followed by 35% of married women. The results concur with studies that were done in Nigeria and DR Congo, that single women and women with higher incomes presented more for PAC compared to married, older, and poorer women [23,24]. However, the study by Erko et al. revealed that married women were 6.7 times more likely to utilize post-abortion family planning than unmarried women [25]. It was thought that married women may be likely to be having sex more regularly than unmarried women, which may explain their high post-abortion family planning utilization [26]. With parity, the results revealed that 39% of women were nullipara. Contrary to the study that was conducted in Zimbabwe, PAC patients were of higher parity women, while nulliparity was common among women seeking treatment for complications of unsafe abortion [27]. More women (48%) were not on any contraceptive methods with only 39% were using injectatbles.

**Table 1:** Demographic profile of participants (n = 23).


CRITERION	CHARACTERISTICS	FREQUENCY	PERCENTAGE %

KZN District	eThekwini district (A):	5	22

	Harry Gwala district (B):	5	22

	uMungundlovu district I:	4	17

	uMzinyathi district (D):	4	17

	King Cetswayo distriI(E):	5	22

Age	15–24	3	13

	25–34	9	39

	35–44	8	35

	45 and above	3	13

Parity	0	9	39

	1	4	17

	2	1	04

	3	02	09

	4 and above	07	30

Marital status	Single	12	52

	Married	08	35

	Divorced	02	09

	Widowed	01	04

Employment status	Unemployed	13	57

	Student/scholar	03	13

	Employed	07	30

Educational level	Primary	02	09

	Secondary	16	69

	Tertiary	05	22

Previous TOP	0	18	78

	1	05	22

Method of contraceptive	None	11	48

	Oral pills	02	9

	Injectables	9	39

	IUCD	1	4


### Themes emerged from raw data

Three themes emerged reflecting challenges from’women’s perspectives on accessing post-abortion care. These themes were: (1) transportation barriers (2) experiences of stigma when seeking PAC, and (3) mistreatment (lack of analgesics, early discharge. Postabortion contraceptive counselling was provided, however, some participants felt it was hurried. The findings were discussed and supported by the literature. Narratives from participants were coded in numbering format and district.

### Theme 1: Transportation barriers

The findings indicate that women always present at the health facilities late. One of the main challenges faced by women who accessed PAC services was the long-distance travelled to access proper care in the hospital. This was further complicated by poor roads and mode of transport in rural areas. This was supported by several respondents, as shown in the following:

**Participant 5 from district A:** *Getting to the hospital took a toll on me, and it was awful. It was raining that day; of all the days, it’s raining. I had to take two buses just to get to that appointment. It was the longest day that I can remember having for a long time*.

**Participant 1 from district D:** *I stay very far from the clinic. I started bleeding while at home early in the morning. My mother-in-law called an ambulance. The ambulance took 3 hours to arrive, and I felt like I was almost dead because I had lost too much blood*.

**Participant 4 from district D:** *I had to travel and change three taxis to come to this hospital, and when I was here, I was given another date to come back again*.

**Participant 2 from district E:** *I took some herbs and started bleeding after a day, and I thought the bleeding would stop. The bleeding went on for four days. I went to the clinic and was told to go to the hospital. I had to travel 259 km to come to this hospital, and when I was there, I was given another date to come back, but I couldn’t return*.

Another challenge cited was the transport from the primary health care facility to the referral hospital. There were no ambulances as cited by:

**Participant 3 from district E:** *I was transferred from the local clinic to this hospital and it was so difficult to get here, I had to wait for three hours for an ambulance to come and collect me*.

The long distance and attitudes of health workers may have played a role in delay and inaccessibility to PAC in KZN.

### Theme 2: Experiences of stigma when seeking PAC

Although abortion is legalized in many developed countries, including South Africa, during data collection, women expressed various ways in which they were being stigmatized for seeking the service in the health institutions. Women reported being labelled as killers, sinners, and mothers of devils. They are told they are a bad influence on the young girls in the community. The following the quotations were cited by participants:

**Participant 5 from district A:** *I was told that I would never have kids in my life because of the abortion, and when I die, the baby will be waiting for me in heaven crying*.

**Participant 5 from district B:** *They see me as a bad girl, and they say that I always sleep around with married men. The other nurse told me I must not associate myself with other young girls because I would teach them how to do an abortion. I felt that I was neglected at the hospital, and I felt like dirt*.

Health care providers’ negative attitudes and behaviours towards a woman who was admitted for post-abortion treatment was very concerning. The quote as cited by one participant was:

**Participant 4 from district E:** *I was admitted with the women who delivered their live babies and the attitude of the health care worker towards me was appalling. The nurse was shouting at me, saying that other women have babies and I have aborted mine. Her words are still haunting me even today*.

**Participant 1 from district C:** *The health care worker threatened to call the police to come and arrest me. “It is a sin. I can’t help you because backstreet abortion is against the law*.”

**Participant 3 from district B:** *When I arrived at the hospital, I was in pain, and I told the clerk at the reception that I was here for post-abortion care, and he told me that I must go to the next window to get the file because he doesn’t deal with abortion women who are killers*.

The findings pointed out that women delayed seeking PAC service even though they were aware of its availability due to fear of stigma and discrimination from the health care workers and the community.

### Theme 3: Mistreatment (lack of analgesics, early discharge)

Women complained that they received no medication for managing the pain during the uterine evacuation and they identified the lack of analgesics as inhuman. The following quotes were cited:

**Participant 4 from district E:** *When I was asked to come in for the procedure, the nurse did not explain anything to me or tell me what she would do. She only asked my age and if I had had an abortion before. No medication was given to me; instead, she put an instrument inside my private part and started cleaning me up. I was screaming, and she kept saying scream like the time when you were sleeping with your boyfriend*.

**Participant 1 from district E:** *I felt horrible pain when the doctor started cleaning my womb. When I told him that I am in pain, he told me you deserve it*.

**Participant 3 from district C:** *I was bleeding profusely and was told that the doctor is still busy with “emergencies,” the doctor came to assist in evacuating left me with the nurse to clean me up. Immediately after the procedure, I was asked to get out of bed, and it was not easy for me to walk out, the pain was too much. I was told to go home and come back after two weeks*.

**Participant 1 from District C:** *They gave me three pills, and then immediately after I swallowed, I started having cramping and heavy bleeding and everything. I was told to go back home because I had cramping and bleeding and was just very uncomfortable. And they said that abortion would take place at home. They did not say anything about me coming back to the hospital for post-abortion care*.

Women were discharged early after evacuation, as hospital beds were insufficient to keep them in the hospital. The quotes below confirm this:

**Participant 3 from District C:** *Because of the shortage of beds, I was discharged on the same day after receiving PAC service*.

**Participant 2 from District E:** *I was surprised when I was discharged from the hospital without any antibiotics or pain killers. When I tried to ask the nurse, she told me that everything was done for me, I can go home*.

Participants voiced their grievances about how they were treated by health care providers when presenting for PAC. Contrary to the earlier quote, one participant received health information from health care providers. The following quotation confirmed this:

**Participant 4 from district E:** *The nurse sat down with me and talked about the dangers of not taking antibiotics and the importance of finishing them to protect myself from the infection*.

However, women who reported spontaneous abortion (miscarriage) were given preference, making women with induced abortion delay seeking PAC services.

## Discussion of findings

Despite evidence that shows that post-abortion care reduces maternal morbidity, mortality, and facilitates the use of contraceptives, the findings revealed barriers experienced when accessing PAC, resulting in a delay in seeking care [28]. In South Africa, induced abortion is permitted on medical or on broader socio-economic grounds, however, women still resort to abortion performed by unskilled providers or in unsafe conditions because of barriers that impede access to safe abortion [29]. Such barriers include distance, lack of information, economic constraints, and lack of confidentiality. Participants who resided very far from the health facilities cited lack of transport as a barrier to access PAC services. Consistent with the findings of this study, poor transportation was identified as a barrier to post-abortion care in Uganda as the geography and geographical barriers had various impacts on access to post-abortion services [30]. Peters et al. and Vlassoff et al. corroborated that increased distance to health facilities and poor local transportation impedes accessibility to primary and secondary health care services [12,31]. Even though the PAC services are free, local transportation results in indirect costs [32,33]. Participants mentioned that hospitals were not having the adequate number of ambulances to fetch women in rural areas, which delays care and leads to PAC complications. The findings were supported by Zaaijman, that the South African Human Rights Commission highlighted the poor health service delivery in rural areas, like insufficient ambulance services [34]. It also stressed that poor road conditions in remote areas further lead to inaccessibility even if an ambulance were available. The lack of adequate and reliable transport was a significant impediment to rural healthcare delivery, delayed health care provision, and worsened people’s health [12]. Women had rights to access safe and legal abortion, however, CTOPA, 1996 states the conscientious objection of providers who do not wish to perform abortions is supported by the constitutional rights of all South Africans to freedom of thought, belief and opinion [14]. The Act further indicates that providers who refuse to perform an abortion must give patients accurate abortion related information of the relevant provider. When the legal abortion services are not assessible or available, women seek help outside the established legal health system, and that brought serious implications on women’s reproductive health and well-being [35]. Jones and Jerman found that of those who provided abortion services, 96% do so at eight weeks of gestation [36]. Accessing a legal abortion provider is a challenge to many women, which may explain why abortion rates have declined. More women have greater access to physicians and the posters on the street poles advertising abortion services than they do to clinics, yet few doctors perform the procedure due to lack of training, disinterest, and controversy surrounding the topic.

The study’s findings were consistent with Gebreselassie and Faundes et al. who found that women tend to be reluctant to seek post-abortion care due to fear of ill-treatment from the health care workers, especially if they have undergone an illegal abortion [16,37]. Tagoe-Darko also confirmed that women might hesitate for fear of experiencing disrespect or abuse from providers [38]. The women were cited to have experienced emotional, physical, and psychological trauma. Most health care workers demonstrated negative perceptions toward PAC services, stating their religious beliefs or conscientious objections [39]. The attitudes of health care workers were cited as the barrier to accessing the PAC services. Most staff members were not willing to provide care and to share information pertaining to abortion services and this lack of information lead inaccessibility to PAC services, delay and to complications. Stigma and discrimination were experienced by participants. Quinn and Chaudior found that women delay seeking post-abortion care if stigmatized because they were protecting themselves from being the victims of discrimination [40]. This was confirmed by Perera et al. and Yegon et al. that stigma and poverty, nonetheless, also played a vital role in influencing the decision-making process [6,41]. Demtsu et al. reported an association between the provision of PAC services, religious, cultural beliefs, morals, and values in terms of care of women seeking PAC services [42].

According to the CTOP Act no 92 of 1996 every woman, regardless of age, race, or background who considers post-abortion care service needs a non-directive professional pre-and post-counseling [14]. Again, according to WHO recommendations, all women should be routinely offered pain medication for example, NSAIDs like diclofenac 25mg thirty minutes before the procedure and ibuprofen 200mg three times a day post-abortion care [1]. However, findings revealed that undergoing an evacuation procedure was a harrowing experience. They were not given any analgesics before the procedure or any pre and post counseling. The discharge was immediately after the evacuation procedure, but participants cited self-care and coping deficits if bleeding and pain doesn’t subside when at home. But this was practised due to a shortage of beds at the facility. Health care workers are expected to ensure that the women are given PAC counselling and family planning before discharge and provide assessment and ongoing referral for more specialist treatment if required. It was also confirmed by Savelieva et al. that family planning counselling contribute to success in pregnancy prevention [43].

## Conclusion

Findings revealed that women who accessed PAC identified a lack of facilities that offered PAC service in rural areas. The long distance, poor road and poor mode of transport were barrier to accessing the services. Findings suggest that while some barriers are not unique to PAC, others may be specific to PAC, especially abortion stigma. Women delayed seeking PAC service even though they were aware of its availability due to fear of stigma and discrimination from the health care workers and the community. The attitudes of health workers may have played a role in delay and inaccessibility to PAC services. Other women complained that they received no medication for managing the pain during the uterine evacuation, and they identified the lack of analgesics to be inhuman. These contributed to inaccessibility to PAC services in the selected health facilities.

## Recommendations

The PAC services should be accessible with the increased number of facilities and adequately trained health care workers with functional equipment and guidelines. Values clarification activities should also address stigma and bias among health workers to ensure respectful care. In addition, health workers should address the abortion stigma among women. Postabortion family planning counseling should be encouraged to provide universal access to postabortion family planning before the woman leaves the facility [44]. Facilitate the establishment of community empowerment activities through community awareness and mobilization [39]. It is essential to disseminate the findings to the Department of Health, Maternal and Child Health Directorate for them to prioritize addressing the barriers to ensure that women have access to this critical life-saving care.

## Data accessibility statement

Some raw data used to support the findings are included in the article can be made available from the corresponding author upon request. This manuscript was derived from the doctoral thesis.

## Abbreviations

PAC – post-abortion care

CTOPA – Choice for Termination of Pregnancy Act
